# Global health education for the post-pandemic years: parity, people, planet, priorities, and practices

**DOI:** 10.1186/s41256-021-00234-y

**Published:** 2022-01-04

**Authors:** Kathryn H. Jacobsen, Caryl E. Waggett

**Affiliations:** 1 Department of Health Studies, University of Richmond, Richmond, VA USA; 2 Department of Global Health Studies, Allegheny College, Meadville, PA USA

**Keywords:** Educational models, Global health, Professional education

## Abstract

Global health degree programs are now offered by institutions of higher education in most world regions. Based on our review of the curricula for many of these programs, we identified five domains that are central to current global health education. “Parity” emphasizes health equity as the ultimate goal of global health. “People” comprises the social, economic, cultural, and political contributors to health and access to medical care for individuals and communities. “Planet” encompasses various aspects of globalization and environmental health that affect population health. “Priorities” and “practices” include the values, data, and tools used to design, implement, and evaluate partnerships, policies, programs, and other global health interventions in countries of all income levels. The pandemic is likely to increase student demand for global health education from the undergraduate through the graduate and professional levels. Our “5 Ps model of global health education” provides a comprehensive framework for the core student learning objectives for global health today. Knowledge of each of these domains is essential for preparing students for meaningful experiential learning and skilled professional practice in global health.

## Background

Global health is an expanding area of academic and professional study in many countries. Many of the leading universities in China have launched degrees in global health during the past decade [1], including studies at the undergraduate level [2]. A growing number of diplomas and degrees in global health are being offered at institutions of higher education in India [3]. In Canada and the United States, a growing number of universities offer master’s degrees with a focus on global health [4, 5] and a rapidly increasing number of colleges and universities offer undergraduate majors and minors in global health [6–8]. Undergraduate and postgraduate degrees are offered by several universities in the United Kingdom [9], and public health training in Europe is globalizing [10, 11]. Increased student demand for global health programs is also occurring in other world regions [12–14].

The core knowledge areas and skills that are featured in academic global health coursework and degree pathways have solidified over the past decade. Most of the global health learning objectives and competencies developed by professional organizations in various world regions over the past decade express similar themes [15, 16], and most of the curricula for global health degree programs at all levels in various countries align with those educational models. For example, we have observed similar trends in the content of introductory undergraduate courses in global health [7] and the content of master’s level curricula in global health in North America [4]. (There is less consistency in how global health education is conceptualized by programs that engage with global health within the context of professional education.)

In our role as the co-chairs of the Consortium of Universities for Global Health (CUGH) Subcommittee on Master’s and Undergraduate Degrees in Global Health (SMUDGH) for the past several years, we have worked with our colleagues to identify global health education programs around the world, including bachelor’s-level programs (such as majors, minors, and undergraduate certificates), master’s and doctoral degrees in global health, and professional degrees (such as master of public health degrees) that offer concentrations in global health as part of professional training in other fields. Most of these programs combine extensive coursework in global health with some type of experiential learning, such as an internship, an international learning trip, or a research project. (By contrast, clinical education programs, such as those in medicine and nursing, typically require little or no formal coursework and instead engage with global health almost exclusively through applied practice experiences in international settings.) We also contributed to the development of a set of 10 CUGH-recommended student learning objectives in global health that align with existing competency models and the most frequently occurring elements of existing academic global health curricula (Table 1) [7]. The learning objectives were vetted by global health educators in several world regions prior to publication in order to confirm that they had international relevance even though they originated from an organization based in North America.

**Table 1:** Consortium of Universities for Global Health (CUGH) recommended student learning objectives in global health [7]

#	Learning OBJECTIVE
1	Describe the history, values, and functions of global health
2	Explain how travel, trade, and other aspects of globalization contribute to health, disease, and health disparities
3	Summarize the social, economic, cultural, and political contributors to individual and population health
4	Examine the connections between human health and environmental health, including considerations of water, sanitation, air quality, urbanization, and ecosystem health
5	Discuss the relationship between human rights and global health
6	Compare the financing and delivery of medical care in countries with different types of health systems and different income levels
7	Evaluate the roles, responsibilities, and relationships of the agencies and organizations involved in financing and implementing public health interventions locally and internationally
8	Compare the burden of disease, disability, and death from infectious diseases, nutritional deficiencies, maternal and perinatal conditions, noncommunicable diseases, mental health disorders, and injuries in countries with different income levels
9	Identify evidence-based, cost-effective, sustainable interventions for promoting health and preventing illness across the lifespan from the prenatal period through older adulthood
10	Apply an interdisciplinary or interprofessional lens to the evaluation of policies and interventions that seek to solve major population health concerns and achieve health equity

The coronavirus pandemic is likely to make some knowledge and competency areas higher priorities for global health curricula in the coming years. In this paper, we introduce our “5 Ps model of global health education,” highlight some of the curricular trends we think are likely to occur as a result of the pandemic, and show how those anticipated areas of increased attention fit within the 5 Ps model.

## The 5 Ps model of global health education

**Fig. 1:**
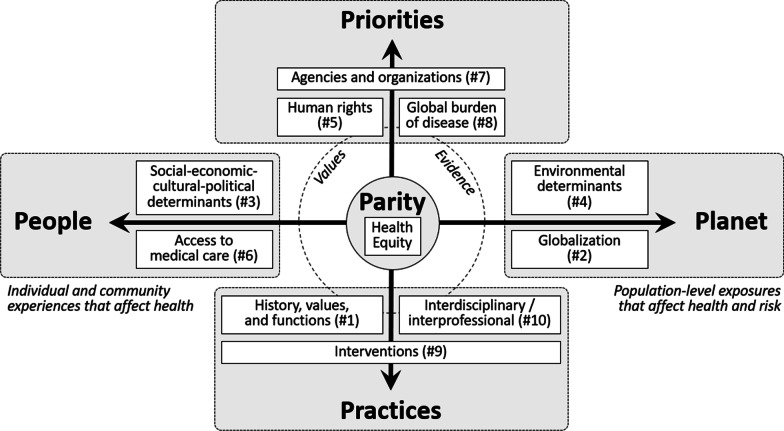
The 5 Ps model of global health education

Based on our analyses of the curricula of academic global health programs in North America and several other regions, we have identified five key current aspects of global health education that in this paper we will refer to as the “5 Ps”: parity, people, planet, priorities, and practices. These can be visualized by placing “parity,” or health equity, at the center of two overlapping axes (Fig. 1). One axis extends from “people” to “planet” to represent the full spectrum of individual, community, national, and global factors that affect human health. The other axis extends from “priorities” to “practices” to represent the full spectrum of policies and interventions that are used to promote health equity and achieve global health goals. The priorities and practices domains both include content related to values (or motivations) and evidence (or research).

Parity is placed at the center of the model because health equity is the central goal of global health. Health equity is the principle that everyone should have an equal opportunity to be as healthy as possible, no matter where that person happens to have been born. While health disparities could technically be reduced by lowering the health status of advantaged populations rather than improving the health status of disadvantaged populations, health equity calls for parity to be achieved by raising the health status of all populations. The COVID-19 pandemic has put a spotlight on some of the ways that global health does not live up to this ideal. Vaccine equity gaps between high- and low-income countries remained sizeable a year after the first coronavirus vaccines were approved for widespread use, even though many high-income countries donated their surplus doses to lower-income partner countries and contributed to COVAX, the collaborative effort by the World Health Organization (WHO), United Nations Children’s Fund (UNICEF), and the Coalition for Epidemic Preparedness Innovations (CEPI) to accelerate global access to coronavirus vaccines [17]. Men have had disproportionately high rates of hospitalization and death from COVID-19, while women have been disproportionately burdened by increased caregiving responsibilities and job loss [18]. The parity domain is also expressed in global health education programs with language related to justice and human rights, and progress toward fully realizing the Universal Declaration of Human Rights (UDHR) has been hampered by the pandemic [19].

The 10 CUGH learning objectives can be used to illustrate the core content for each of the 4 poles of the 5 Ps model [7].

- “People” includes the social, economic, cultural, and political contributors to health as well as the financing and delivery of medical care for individuals and communities.
- “Planet” encompasses various aspects of globalization, such as international trade and travel, as well as the connections between human health and environmental and ecosystem health.
- “Priorities” are expressed through learning objectives related to human rights, ethics, and the values that guide prioritization; comparative health metrics and other types of research that provide evidence for policy development, implementation, and evaluation; and the agencies and organizations that lead the prioritization, funding, and implementation of global health interventions.
- “Practices” incorporates learning objectives that pertain to understanding the history, values, and functions of global health; the interdisciplinary and interprofessional skills that are used to improve health in all populations; and the various types of health interventions that promote health equity across the lifespan.

The verbs used to express the expected level of learning associated with each of the 10 learning objective areas might need to be adjusted for particular courses and programs. For example, undergraduate programs might expect their students to understand theoretical concepts and analyze basic data sets, while postgraduate programs might expect their students to evaluate complex global health challenges and create detailed plans for new global health interventions. However, the general themes apply to all levels of global health education.

## People and planet

The “global” in global health expresses two meanings: worldwide and comprehensive. Global health education programs almost always include in-depth examinations of the social, economic, educational, cultural, policy, demographic, and other factors that affect various facets of health, quality of life, and the ability to access high-quality preventive, diagnostic, therapeutic, and rehabilitative medical care. Public health courses cover many aspects of the “people” dimension, with global public health courses examining these in international context rather than maintaining a predominantly domestic focus. In multidisciplinary global health programs, especially those at the baccalaureate level, this curricular area often includes coursework in medical anthropology, health economics, health psychology, and other social science fields [7]. The most robust “people”-oriented courses include content from countries across the income spectrum rather than focusing solely on health in lower-income countries and disadvantaged populations.

Most global health degree programs currently lean more heavily toward the “people” side of the people–planet axis than the “planet” side, but concerns about the anticipated adverse effects of global climate change on human health and fears about zoonotic pathogens sparking future human pandemics are drawing many programs closer to the center of the axis. Global climate change is likely to become the dominant global health concern of the twenty-first century as extreme temperature and weather events become more frequent and widespread [20, 21]. Traditional environmental health policies and practices tended to emphasize the ways that natural resources and the natural and built environments can be used for the benefit of humans. In contrast, the recently established planetary health movement argues that humans need to care for the planet because the health of future generations of humans is wholly dependent on a healthy Earth [22]. Globalization processes like international trade have contributed to global climate change, and they have also made it easier for local infectious disease outbreaks to become global threats. The coronavirus pandemic has made the world much more aware of the threat posed by spillover events in which wildlife infections cross into human populations [23]. As global health curricula are revised over the next few years, they are likely to integrate more content on climate change and planetary health [24] and on pandemic preparedness and other forms of emergency management [25].

## Priorities and practices

Global health partnerships generally achieve the best outcomes when the collaborators make decisions that are grounded in shared values, based on scientific evidence, and respectful of diverse geopolitical and other perspectives [26]. One way to interpret the priorities–practices axis is to view “priorities” as being about funders and the ways they make decisions about which policies and partnerships they will support while “practices” is about implementers and the methods they draw on when they design, implement, and evaluate programs and projects. Health equity and human rights are guiding principles for both funding and implementation, and funders and implementers both tend to value actions that benefit large populations, promote international cooperation, respond to humanitarian concerns, and support economic and political security [27, 28]. The evidence for both funders and implementers include comparative metrics, such as those related to the Sustainable Development Goals (SDGs) and the global burden of disease, as well as quantitative and qualitative research findings from a variety of disciplines.

Older models of international health typically featured high-income donor countries setting the agenda for the health projects they supported in lower-income recipient countries [29]. Modern global health aspires to have more transparent and equitable processes for establishing priorities [30]. The “priorities” domain of global health education develops students’ knowledge of the players involved in setting global health priorities and the global health agencies, organizations, roles, relationships, policies, laws, regulations, governance, and resources that are involved in that process. Current coursework related to this domain typically focuses on global health systems and policies [4]. Most global health education programs also require some training in epidemiology, statistics, and research methods [4, 7]. The pandemic may increase demand for courses on comparative health metrics and other data science applications that facilitate decision-making [31]. There may also be greater demand for courses on global health law, such as courses that focus on human rights law, including laws pertaining to access to essential medicines and vaccines, and courses that examine the methods for negotiating, implementing, and enforcing health treaties like the International Health Regulations (IHR) and the WHO Framework Convention on Tobacco Control (FCTC) [32].

The “practices” domain focuses on applied global health interventions and the processes by which they are implemented. Coursework in this area often features case studies of historic and recent global health initiatives that support development of applied program management skills. Global health education programs often embed professional competencies related to cross-cultural communication and leadership within coursework in this area, since rising global health leaders will be most effective in the workforce when they are skilled communicators who demonstrate cultural awareness, are capable of motivating others to achieve a shared set of goals, and have the flexibility to adapt methods to fit with evolving realities [33–35]. Global health training is often rooted in clinical practice (such as medicine or nursing) or community practice (such as public health, law, or social work), but comprehensive global health education for the post-pandemic era will benefit from the inclusion of perspectives from additional sectors as well as from examinations of best practices for interdisciplinary, interprofessional, and intersectoral teamwork.

## Curricular implications

While the 5 Ps model is intended to highlight the core curricular areas of academic global health degree programs, it also points toward gaps in some types of clinical and professional global health education. The dominant models of global health engagement in various professions vary considerably and do not always connect with both the global and the holistic aspects of global health. For example, medical education about global health often focuses almost exclusively on cross-cultural clinical experiences, even though that model has been criticized for perpetuating inequities rooted in colonialism [36, 37]. Medical global health embraces health equity as a principle and invests heartily in the practice domain, but it rarely engages deeply with the people–planet axis. Public health concentrations in global health may also weight the practice domain much more heavily than other poles. For example, the Association of Schools and Programs of Public Health (ASPPH), which is based in the United States but has members from several world regions, recommends six competencies for Master of Public Health (MPH) concentrations in global public health, and all focus on applied practice skills such as monitoring and evaluation, multisectoral interventions, sustainable workforce development, and critical self-reflection [5].

As curricula for courses, degrees, and other educational programs are updated to meet emerging needs revealed by the COVID-19 pandemic, the 5 Ps may provide a tool for confirming that plans for updated course and program content retain the balance or weighting that is desired by program faculty. Some programs will opt to expand their engagement with planetary health in order to achieve a better balance between the “people” and “planet” poles. Some will incorporate more “theory” into their programs so that they are not too heavily weighted toward the “practices” pole. Given the growing recognition that power hierarchies and nonrepresentative leadership adversely affect all aspects of global health, including global health education [26, 38, 39], some programs will invest more resources in making global health degrees and mentorship more accessible to and inclusive of students who are from population groups that are currently under-represented in global health leadership.

## Global health education for professional practice

Effective global health work is grounded in a strong understanding of the values of the field and knowledge about core principles and major practice areas. Everyone working in global health should be familiar with the history, values, and functions of global health; the ways that globalization shapes individual and population health; the major social, economic, cultural, and political determinants to health; the links between human health and environmental health; the relationship between health and human rights; the various models for financing and delivering medical care; the entities that are involved in global public health financing, implementation, and governance; the major causes of disease, disability, and death in various populations; and the wide variety of interventions that improve population health; and all global health professionals should have the ability to design and evaluate innovative solutions to pressing global health issues [7].

The pandemic is likely to increase student interest in global health and demand for global health education from the undergraduate through the graduate and professional levels. Some learners will opt to make global health their primary area of study, while others will pursue global health education as a secondary field, as an application of a primary discipline, or through certificates, service-learning projects, study abroad, research, and other types of experiential learning that complement training in medicine, public health, law, environmental science, urban planning, and other primary fields. As global health continues to mature as a discipline, it will be valuable to define the specific knowledge and skill sets that are priorities for different levels and types of education. It will also be useful for countries to identify the optimal ways to articulate global health curricula at the bachelor’s, master’s, and doctoral levels within their educational systems so that duplication of content across levels is reduced and each level builds on previous levels of study.

Practical experience in international settings is not sufficient preparation for global health work. No matter what area of specialization a rising global health professional plans to pursue, profession-specific training in global health must build on education that is specific to global health. The 5 Ps model provides a framework for confirming that global health programs, courses, and degree pathways are equipping learners with a comprehensive foundation of global health knowledge.

## Data Availability

Not applicable.

## Abbreviations

5 Ps: Parity, people, planet, priorities, and practices
ASPPH: Association of Schools and Programs of Public Health
CEPI: Coalition for Epidemic Preparedness Innovations
COVAX: The COVID-19 Vaccines Global Access Facility
CUGH: Consortium for Universities of Global Health
FCTC: WHO Framework Convention on Tobacco Control
IHR: International Health Regulations
MPH: Master of Public Health
SDGs: Sustainable Development Goals
SMUDGH: CUGH Subcommittee on Master’s and Undergraduate Degrees in Global Health
UDHR: Universal Declaration of Human Rights
UNICEF: United Nations Children’s Fund
WHO: World Health Organization
